# Identifying electron transfer coordinates in donor-bridge-acceptor systems using mode projection analysis

**DOI:** 10.1038/ncomms14554

**Published:** 2017-02-24

**Authors:** Xunmo Yang, Theo Keane, Milan Delor, Anthony J. H. M. Meijer, Julia Weinstein, Eric R. Bittner

**Affiliations:** 1 Department of Chemistry, University of Houston, Houston, Texas 77204, USA; 2 Department of Chemistry, University of Sheffield, Sheffield S3 7HF, UK; 3 Department of Physics, University of Houston, Houston, Texas 77204, USA

## Abstract

We report upon an analysis of the vibrational modes that couple and drive the state-to-state electronic transfer branching ratios in a model donor-bridge-acceptor system consisting of a phenothiazine-based donor linked to a naphthalene-monoimide acceptor via a platinum-acetylide bridging unit. Our analysis is based upon an iterative Lanczos search algorithm that finds superpositions of vibronic modes that optimize the electron/nuclear coupling using input from excited-state quantum chemical methods. Our results indicate that the electron transfer reaction coordinates between a triplet charge-transfer state and lower lying charge-separated and localized excitonic states are dominated by asymmetric and symmetric modes of the acetylene groups on either side of the central atom in this system. In particular, we find that while a nearly symmetric mode couples both the charge-separation and charge-recombination transitions more or less equally, the coupling along an asymmetric mode is far greater suggesting that IR excitation of the acetylene modes preferentially enhances charge-recombination transition relative to charge-separation.

## Abstract (web summary)

Manipulating chemical reactions using laser pulses to control electron transfer is an attractive goal, however much of the underlying physics remains unexplored. Here the authors analyse and explain the intramolecular electronic transfer occurring during charge-separation in acetylene, a model donor-bridge-acceptor molecule.

One of the highly desirable goals of modern chemical physics is to directly manipulate the outcome of a chemical reaction using ultrashort laser pulses. The absorption of a visible or ultraviolet photon by a molecule initiates a cascade of events driven by a sudden change in the electronic state followed by nuclear motion, in which the excess vibrational energy is rapidly dissipated and redistributed. One suggested means of controlling the outcome of an electron transfer process is to use an infra-red light to selectively excite specific nuclear motions that are strongly coupled to an electron transfer event^1^,^2^,^3^,^4^.

In this context, dynamics of photoinduced electron transfer have been investigated in a series of three donor-bridge-acceptor molecular triads^5^,^6^,^7^. The triads consist of a phenothiazine-based (PTZ) donor linked to a naphthalene-monoimide (NAP) acceptor via a Pt-acetylide bridging unit^7^. The structures of the triads are given in Fig. 1a. All three systems undergo a similar sequence of processes after following ultraviolet excitation: electron transfer from the Pt-acetylide centre to the NAP acceptor, resulting in a charge-transfer state, *D*−*B*^+^−*A*^−^, which due to strong spin–orbit coupling efficiently populates triplet charge-transfer state, CT. Further electron transfer leads to a fully charge-separated state (CSS) *D*^+^−*B*−*A*^−^ with the electron and hole localized on the acceptor and donor units, respectively. The charge-transfer state can also undergo charge recombination to form a localized triplet exciton on the NAP unit (^3^NAP), or the ground state. Both CSS and ^3^NAP decay to the singlet ground state on the nanoseconds and sub-millisecond time scales, respectively. We also show in Fig. 1b the triplet energy along a linear interpolation coordinate connecting the ^3^NAP minimum energy geometry to the CT minimum energy geometry. Between the two is a significant energy barrier reflecting the relative rotation of the NAP and the PTZ groups about the CC-Pt-CC axis.

The ultraviolet pump-IR push experiments performed on these triads showed that infrared (IR) excitation of bridge vibrations after the initial ultraviolet pump radically changes yields of intermediate states. Excitation of the -CC-Pt-CC- localized vibrations in the CT state of PTZ-complex **2** at 1 ps after the ultraviolet pump decreases the yield of the CSS state, whilst increasing that of the ^3^NAP state. IR excitation in the course of electron transfer has caused a 100% decrease in the CSS yield in **1**, ∼50% effect in **2** and no effect in **3**. This demonstration of control over excited-state dynamics strongly suggests that the acetylide stretching modes are significantly involved in the electron/nuclear coupling in these systems and play central roles in the electron transfer process.

This paper investigates the excited-state dynamics in such systems with the goal of understanding how specific vibrational modes can dramatically influence the state-to-state dynamics. To do so, we employ a mode projection method we have developed recently that efficiently determines a minimal set of vibrational normal modes that optimize the electronic coupling between two electronic states. We combine this approach with a time-convolutionless master equation (TCLME) method for computing state-to-state rate constants. The approach has been benchmarked and used for many systems, ranging from organic photovoltaics to the molecules in Closs' classical experiments^8^,^9^,^10^,^11^,^12^,^13^,^14^. Besides the computation of transfer rate constant, our method includes a mode projection scheme, which can parse out a hierarchy of nuclear motions primarily coupled to the transition. The most important vibrational motion is termed ‘primary mode' in our previous work, as a Lanczos algorithm is employed in the projection^13^,^14^. Our results indicate that the majority of the electronic coupling is mediated by local acetylide stretching modes.

The structure of this paper is as follows. We first review our theoretical approach which connects an *ab initio* determination of all input parameters to the state-to-state dynamics. We then present the results of these investigations and discuss their implications in interpreting the IR control experiments discussed above. We focus on compound **2** as best illustrating the competition between the two pathways of electron transfer.

## Results

### Quantum chemical workflow

**Figure 2:**
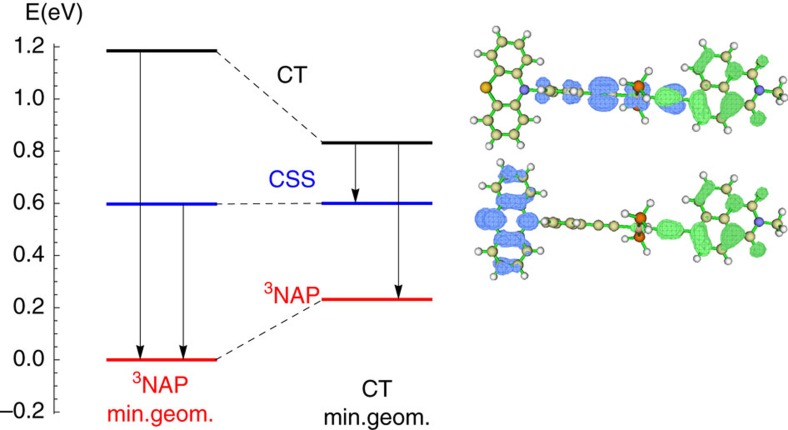
Energy level diagram for the triplet states of PTZ at the ^3^NAP and CT state geometries. The electron/hole distributions for the CT and CSS are shown to the right (green=electron, blue=hole).

In this paper, we focus our attention on the PTZ system and anticipate that the other systems in this study will exhibit similar behaviour due to the overall similarity of the various donor groups. For purposes of facilitating the calculations, the molecular structures are simplified such that the P(Bu)_3_ moieties and octyl chain of the NAP group were truncated to -PH_3_ and a single methyl group, respectively. In all quantum chemical calculations, we used the B3LYP functional Stuttgart/Dresden (SDD) pseudo-potential and associated valence basis set for Pt and 6-31G(d,p) for the other atoms. We also used the polarizable continuum model to account for the dichloromethane solvent^15^,^16^ as per from refs 5, 6, 7. The transition dipole moments and electron/hole distributions surfaces were calculated using the Multiwfn (v3.3.8) programme^17^. An energy level diagram based on our calculations is sketched in Fig. 2a together with the corresponding electron/hole distribution plots. To obtain the diabatic potentials and couplings, we perform a geometry optimization of both the lowest triplet (^3^NAP) and the third triplet excited states (CT). As discussed below, we use the optimized states as reference geometries for determining the diabatic coupling within the Generalized Mulliken Hush (GMH) approximation^18^,^19^. The normal modes and vibrational frequencies were obtained by harmonic expansion of the energy about the CT state. Once we have determined the diabatic states and couplings, we use the TCLME approach from ref. 20 to compute the time-correlation functions and state-to-state golden-rule rates. We also use the projection technique to determine an optimal set of normal modes and determine the number of such optimal modes that are required to converge the time-correlation functions to a desired degree of accuracy. We then use both the CT and ^3^NAP minima as reference states for computing the diabatic potentials and couplings necessary for computing rates and modes. Those obtained at the CT minimum can be used to compute transitions originating in from the CT state, while those obtained at the ^3^NAP minimum can be used for transitions terminating in the ^3^NAP state.

### Primitive model

The primitive model consists of a system with localized electronic states, |*a*〉 and |*b*〉 and a set of internal vibrational modes, **q**.


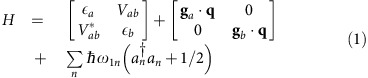


where 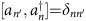. We shall refer to these modes as the primitive modes. Also we have adopted a compact notation for the linear electron–phonon coupling


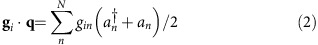


where **g**_*i*_ are the linear force vectors acting on the phonon modes. Diagonalizing just the electronic part (at the initial geometry), our model produces a set of vertical excitation energies,


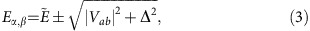


where 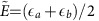 and 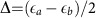 and tan 2*θ*=|*V*_*ab*_|/Δ is the mixing angle between the local electronic states.

In practice, we construct this Hamiltonian using input from quantum chemistry. The electronic Hamiltonian is referenced to a localized set of adiabatic states determined at an initial starting geometry, typically taken to be at the energy minimum of either the initial or final states, in which case we can set either **g**_*a*_=0 or **g**_*b*_=0. In other words, we can obtain *E*_*α*,*β*_ directly from quantum chemistry and need to determine the diabatic energies 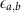 and the off-diagonal diabatic coupling *V*_*ab*_. To do so, we use either the Edmiston-Ruendenberg (ER) localization scheme or GMH approach to approximate the donor and acceptor states^18^,^19^. GMH works well for linear or near-linear systems, but does not generalize well to non-collinear systems with multiple charge centres. ER localization can be seen as an extension of GMH which overcomes some drawbacks of GMH^21^. The molecular systems here are nearly linear and the charge centres generally along the C≡C axis. As the result, both the GMH and ER localization approaches are expected to give similar results. The diabatic coupling given by GMH is


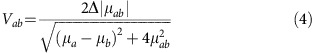


where *μ*_*a*_ and *μ*_*b*_ are the dipole moments of corresponding adiabatic states, and *μ*_*ab*_ is the transition dipole moment between two states. Note, that *V*_*ab*_ is also related to the adiabatic energy difference and mixing angle via *V*_*ab*_=(*E*_*α*_−*E*_*β*_)sin(2*θ*)/2.

### Computing spectral densities and transition rates

Once a suitable set of parameters and vibrational modes have been determined, we construct the spectral density in terms of the electron–phonon coupling operators between states *n* and *m*


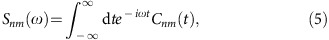


where


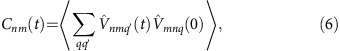


is the autocorrelation function of the polaron-transformed electron–phonon coupling operator in the Heisenberg representation and 〈⋯〉 denotes a thermal average over the vibrational degrees of freedom. The derivation and explicit form for the kernel in equation (5) is quite lengthy and is given in ref. 20. It is important to notice that *C*_*nm*_(*t*) includes scattering between different phonon modes *q*→*q*′ as the result of the electronic transition. Using this approach, the golden-rule rates are given by


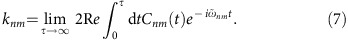


where 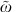 is the transition frequency. In a practical sense, we take *τ* to be some finite time at which the autocorrelation function *C*_*nm*_(*t*) has relaxed to zero. In our numerical studies, we use the *C*_*nm*_(*t*) to benchmark the convergence of our model with respect to the number of nuclear modes.

### Primary modes

Transforming the electron/phonon coupling to the non-local representation results in a new operator with both diagonal (longitudinal) and off-diagonal (transverse) components: The same unitary transformation that diagonalizes the electronic part of the Hamitonian mixes the force vectors **g**_1_ and **g**_2_. Since this is a linear transformation, we define new non-orthogonal coordinates and forces that describe the electron/phonon coupling. Operationally, these are taken as vectors along each of the normal vibrational modes. By analysing such forces we can gain insight into the dynamics of the transition as well as open avenues for developing improved approximations for the state-to-state transition rates. In our previous works^13^, we presented a Lanczos-based ranking algorithm that projects out a ranked set of orthogonal nuclear modes that optimally capture the electron/nuclear couplings. We refer to the highest-ranked mode identified by the algorithm as the ‘primary Lanczos mode' (PLM).

For our purposes, an ‘exact' calculation involves including all nuclear vibrational modes. In our previous work we showed that both *C*(*t*) and the total transfer rate constant, *k*_*nm*_ calculated using only the first few projected modes provide an excellent agreement with the exact quantities computed using the full set of normal modes, as well as the experimental rates, when parameterized using accurate quantum chemical data^13^. Since this is a non-standard technique, we include in the Supplementary Information (SI) a set of Mathematica scripts for constructing the Lanczos modes.

### Marcus theory rates

By far the simplest approach for computing the transition rates is using the Marcus expression for the electron transfer rate constant,


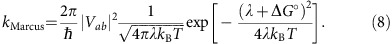


Table 1 provides a summary of the various parameters we compute for various transitions in the PTZ system. The two columns under the heading labelled ^3^NAP correspond to parameters computed using the ^3^NAP minimum as a reference geometry while those under the heading labelled CT correspond to parameters computed using the CT reference geometry. A careful examination of this table portends a difficulty in using the ^3^NAP geometry as a reference. In fact, for the CSS→^3^NAP, the driving force is in the wrong direction since the derived CSS state lies lower in energy than the ^3^NAP state, which is inconsistent with the experimental observations.

### Time-convolutionless master equation rates

**Figure 3:**
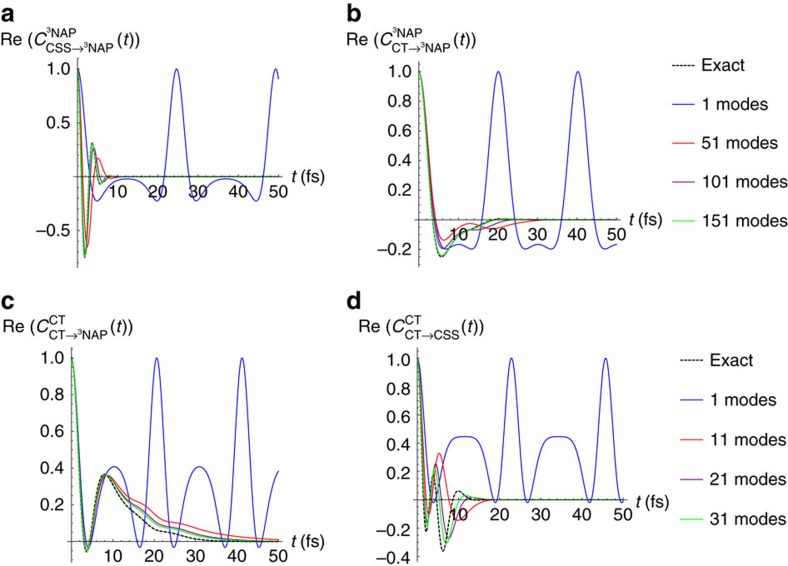
Convergence of electronic correlation for specified transitions with respect to projected modes. The `exact' result corresponds to using all modes. (**a**) CSS→^3^NAP at ^3^NAP geometry, (**b**) CT→^3^NAP at ^3^NAP geometry, (**c**) CT→^3^NAP at CT geometry and (**d**) CT→CSS at CT geometry. (For electronic correlation functions see equation 6).

To compute the rates using the TCLME expression (equation (7)), we begin by computing the electron/nuclear correlation function and compare its convergence with respect to the number of Lanczos modes. Recall that the Lanczos modes are determined by an iterative ranking algorithm that identifies superpositions of normal modes that optimize the electron/phonon coupling. Figure 3 gives a summary of these numerical tests in which we compute *C*_*nm*_(*t*) versus time with an increasing number of Lanczos modes. In all cases, we compare to the ‘exact' result in which all nuclear modes were used. The top two figures (Fig. 3a,b) use the ^3^NAP as the reference geometry. In these cases, convergence of *C*_*nm*_(*t*) with respect to the number of modes proved to be problematic for both transitions considered. Correspondingly, the rates computed using this geometry also compare poorly against the observed experimental rates, although are an order of magnitude closer than Marcus rates. We speculate that this may signal a break-down in the Condon approximation which insures separability between nuclear and electronic degrees of freedom.

In Table 2, we summarize both the experimental and computed state-to-state rates for the PTZ system. Given the complexity and size of the system, overall the numerical rates computed using the exact TCLME approach are in quantitative agreement with the experimental rates, particularly for those using the CT geometry as a reference point (cf, Fig. 3c,d). We note that fewer projected modes (30–50) are needed to converge the correlation function out to the first 50 fs when using the CT geometry. Furthermore, while Marcus rate for the CT→CSS transition agrees with the exact TCLME result, it misses the CT→^3^NAP experimental rate by 4 orders of magnitude whereas the TCLME rate is in much better agreement with the experimental rate.

If we compare the exact TCLME rate, which uses the full set of normal modes in constructing the *C*_*nm*_(*t*) correlation function, to the rate computed used only the PLM (TCLME+PLM), for both the CT→CSS and CT→^3^NAP rates, the single mode approximation is within 86% of the exact result. This indicates that while multiple vibrational normal modes contribute to the electronic coupling, the linear combination identified by the projection algorithm carries the vast majority of the electron/phonon coupling. This is entirely consistent with our previous study of triplet energy transfer in small donor-bridge-acceptor systems^13^,^14^.

### Breakdown of the condon approximation

**Figure 4:**
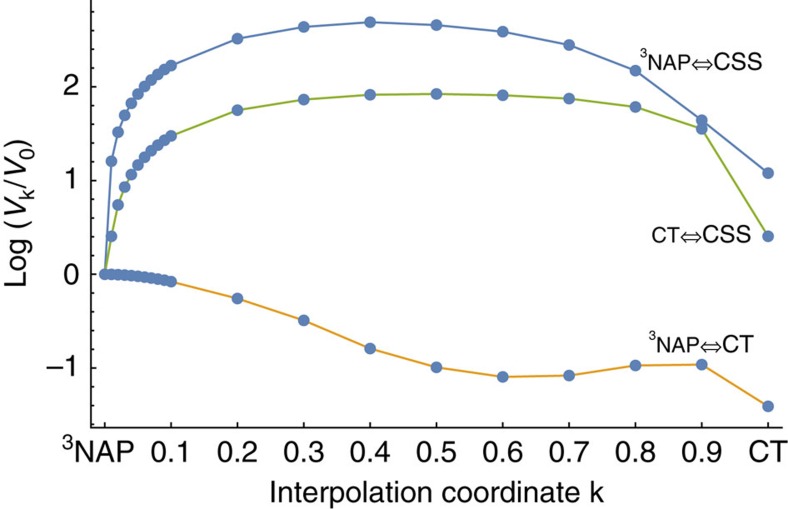
Diabatic couplings. Diabatic couplings computed along a linear interpolation coordinate connecting the ^3^NAP→CT optimized geometries.

Our theoretical model explicitly relies on two assumptions: that the excited-state surfaces are nearly parabolic and we can separate nuclear and electronic motion via the Condon approximation. The first assumption holds since the excited-state potentials are parabolic along C≡C stretching coordinate as shown in refs 5, 6. The latter assumption implies that the diabatic coupling is independent of nuclear coordinates, that is, our parameters do not change significantly with geometric changes in the molecular shape. In Fig. 4 we plot the GMH diabatic coupling along an interpolation coordinate connecting the ^3^NAP equilibrium geometry to the CT equilibrium geometry. It is clear that diabatic couplings undergo significant changes (note the *y* axis is logarithmic), especially in the CSS →^3^NAP case, where the coupling changes by over two orders of magnitude with only a small change in the nuclear geometry.

To explore the effect of the change of diabatic coupling, we substituted the original coupling with an average coupling, 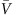, computed along the interpolation coordinate. The correction brings significant improvements, to both the Marcus and TCLME approaches, at the ^3^NAP geometry, especially for the problematic CSS→^3^NAP case where the discrepancy between the theoretical and experimental rates is reduced significantly. In fact, using the averaged coupling brings the TCLME rates into near perfect agreement with the experimental rates.

However, changing how the diabatic coupling is determined will affect the Δ in equation (3) and hence the computed driving force Δ*G*° used in the Marcus expression since


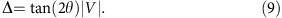


For the CSS→^3^NAP transition at the ^3^NAP geometry, Δ*G*° is insensitive to whether *V* or 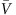 is used even though 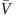 is 2 orders of magnitude greater than diabatic coupling computed at the ^3^NAP geometry. The reason for this is that even though the diabatic coupling changes considerably, the mixing angle between the diabatic and adiabatic representations changes only slightly. On the other hand for the CT→^3^NAP transition at the ^3^NAP geometry, the diabatic coupling is much larger and 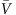 is on the same order of magnitude as the original coupling. Here, we see that the driving force obtained using the averaged coupling 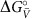
=−0.851 eV is much closer to the electronic energy difference between the two optimized states (0.818 eV).

However, for transitions originating from the CT geometry, using an averaged coupling does not lead to quantitative improvement of the rates, most markedly for the CT→CSS transition. In fact in this case, the average coupling 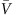
>(*E*_*α*_−*E*_*β*_)/2 in equation (3) and produces an physically unreasonable mixing angle between the adiabatic states. Looking at Fig. 4, we suspect that the best choice of diabatic coupling is where the coupling varies little with molecular geometry.

### Primary mode analysis

As discussed earlier, our ranking algorithm allows us to rapidly determine the vibrational motions that optimize the electron/nuclear couplings. In addition to providing an accurate way to compute rate constants, they provide additional insight into actual dynamics. Here, we shall focus upon the transitions originating from the CT geometry. Generally speaking, the highest-ranked mode, termed the PLM, captures much of the short-time dynamics of the transitions. We can see this by looking at the *C*_*ab*_(*t*) correlation functions in Fig. 3 where the primary mode reproduces the exact dynamics up until the first recurrence at ≈10 fs. Adding more modes rapidly improves agreement out to longer and longer times; however, the majority of the coupling is actually concentrated in the PLM.

**Figure 5:**
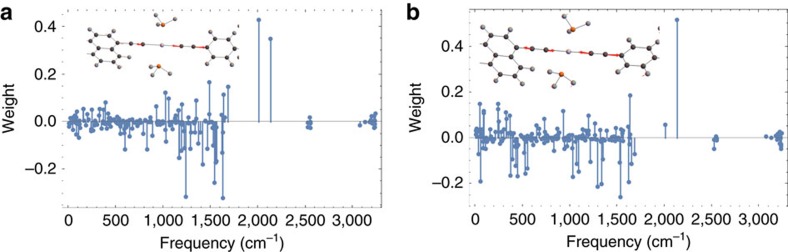
Projection of primary mode on normal vibrational modes. (**a**) CT→^3^NAP, and (**b**) CT→CSS calculated at CT geometry onto the normal modes of CT. Embedded molecule shows the atomic displacement vectors of primary mode.

**Figure 6:**
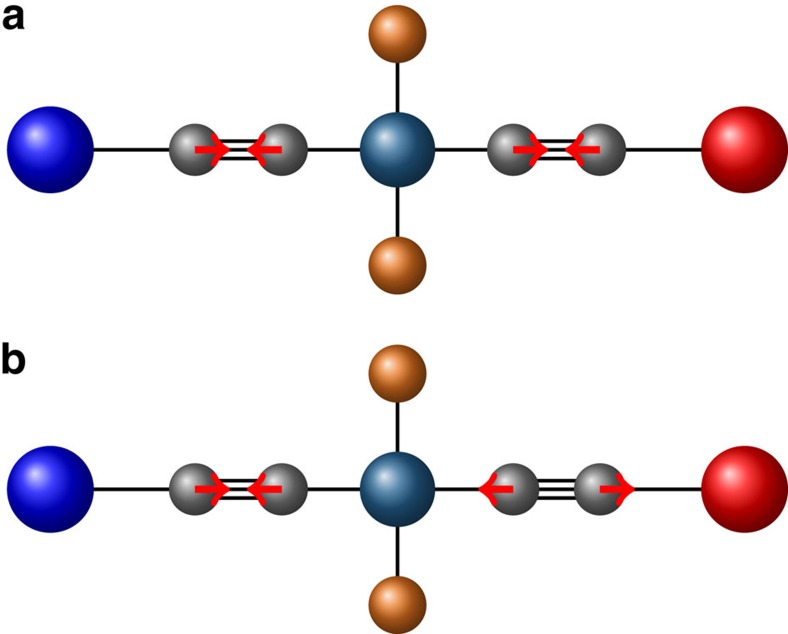
Acetylene bond-stretching motions. Symmetric (**a**) and anti-symmetric (**b**) stretch.

In Fig. 5 we show PLMs for the CT→^3^NAP and CT→CSS transitions as projected onto the normal modes of the full molecule. In all cases, the PLM is composed of a linear combination of normal modes spanning the entire range of molecular motions. Here, most of the projection is concentrated upon the higher frequency modes involving the acetylene stretching modes which confirms our initial suspicion that these stretching modes are strongly coupled to the charge-transfer dynamics. However, while both transitions involve acetylene bond-stretching motions, the CT→CSS transition involves a symmetric combination, whereas the CT→^3^NAP involves both the symmetric and anti-symmetric combination, as shown in Fig. 6.

It is tempting to conclude that the secondary IR push used in the experiments preferentially excites the asymmetric mode and thus selectively enhances the CT→^3^NAP transition. Indeed, from our calculations, the IR oscillator strength of the asymmetric mode is roughly an order of magnitude greater than the symmetric mode. It agrees to the fact that in experiment, the asymmetric normal mode extinction coefficient is 3 times larger than that for the symmetric normal mode. In the CT→^3^NAP, both types of acetylene stretching motions (symmetric and asymmetric) contribute more or less equally to the electronic coupling while in the CT→CSS transition, only the symmetric acetylene motion carries the majority of the coupling. Consequently, driving these modes with the IR pulse increases the electronic coupling, consistent with the experimental observation that IR excitation following formation of the CT states accelerates the CT→^3^NAP transition relative to the CT→CSS transition. This claim can be justified theoretically by examining how the vibrational population of a given normal mode enters into the transition rate equation in equation (7), which we give in the ‘Methods' section.

## Discussion

We present here an analysis of the vibrational modes that couple to, and drive, the state-to-state electronic transitions, and largely determines their relative efficiencies of electron transfer in a model Donor-Bridge-Acceptor system, where the -CC-Pt-CC- unit acts as a bridge. Our analysis is based upon an iterative Lanczos search algorithm that finds superpositions of vibronic modes that optimize the electron/nuclear coupling using input from *ab initio* excited-state quantum chemical methods. Our results indicate that the electron transfer coordinate which corresponds to electronic transition between the branching charge-transfer state and CSS; or between the CT state and the acceptor-localized triplet state (3NAP), can be dominated by symmetric and anti-symmetric stretching vibrations involving two acetylide groups on both sides of the Pt centre. We show that the relative magnitude of coupling between the CT and the CSS states versus that between the CT and 3NAP states largely determines the outcome of the IR push. In particular, the relative magnitude of coupling between the CT and the CSS state versus that between the CT and the ^3^NAP states largely determine the outcome of the IR push. In particular, while the symmetric mode couples both the CT→^3^NAP and CT→CSS transitions more or less equally, the coupling along asymmetric mode is far greater in the CT→^3^NAP transition. This analysis is consistent with recent optical control experiments on these molecular units reported in refs 5, 6, 7.

The proposed analysis, which is described in technical detail in the ‘Methods' section, allows one to identify reaction coordinates in ultrafast transitions between coupled electronic states. The results agree with the experimental observations, and identify the reaction coordinate for the electron transfer process in the model system. The algorithm illustrated here in the example of photoinduced charge-transfer may be of considerable utility for understanding of a multitude of light-induced reactions where several electronic states are involved in ultrafast transformations.

## Methods

### Electron/phonon Hamiltonian

We present here a recapitulation of the electron/phonon coupling model and derivation of the golden-rule rates from ref. 20. Our analysis begins by writing a general Hamiltonian describing the coupling between a set of discrete electronic states 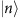 with a set of phonon oscillators. Assuming that the coupling is linear on *both* the diagonal and off-diagonal, one obtains (with *ħ*=1)


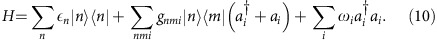


Here |*n*〉's denote electronic states with vertical energies 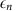, 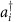 and *a*_*i*_ are the creation and annihilation operators for the normal mode *i* with frequency *ω*_*i*_, and *g*_*nmi*_ are the coupling parameters of the electron–phonon interaction which we take to be linear in the phonon normal mode displacement coordinate. Parameters for this model *H* can be obtained from electronic structure calculations as described in the manuscript and in our recent publications^13^,^14^. Code for extracting the necessary information and performing the analysis are included in the Supplementary Information.

We can separate *H* into a part that is diagonal with respect to the electronic degrees of freedom,


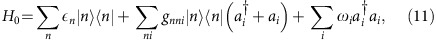


and an off-diagonal part *V*


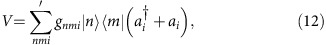


where the prime at the summation sign indicates that the terms with *n*=*m* are excluded. This separation is useful for the following two reasons. First, in many systems only off-diagonal coefficients *g*_*nmi*_ are small compared to *g*_*nni*_ Hence, *V* can be treated as a perturbation. Second, for many cases of interest, the initial density matrix commutes with *H*_0_. In this case, the separation gives simpler forms of the master equations.

A crucial component of our analysis is to diagonalize *H*_0_ with respect to the normal mode degrees of freedom. This is achieved with the unitary shift operator (polaron transformation)


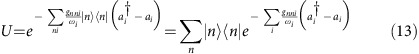


as


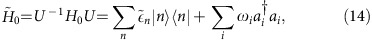


where the renormalized electronic energies are


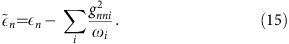


Applying the polaron transformation to *V* gives


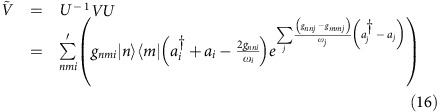


We now introduce dressed operators


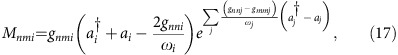


we can rewrite 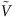 in a compact form as


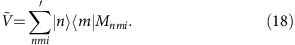


The terms involving (*g*_*nnj*_−*g*_*mmj*_)/*ω*_*j*_ can be related to the reorganization energy


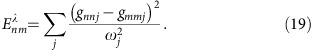


Similarly the energy difference between the renormalized energies is related to the driving force


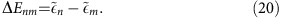


As can be seen from equation (16), in the transformed picture the electronic transitions from state |*n*〉 to |*m*〉 are accompanied not only by the creation or annihilation of a single phonon of mode *i* but also by the displacements of all the normal modes.

### Golden-rule rate expression

Pereverzev and Bittner (ref. 20) also give the Fermi Golden-rule rates for transitions between states *m* and *n* as in terms of the autocorrelation function of their time-dependent coupling


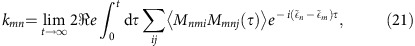


where


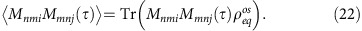


Due to the explicit form of operators *M*_*nmi*_ (equation (17)) the calculation of the correlation functions in equation (22) can be reduced to the averaging of the displacement operators over the equilibrium ensemble. After straightforward but lengthy calculations, one can arrive at a compact expression for the autocorrelation function of the electron/phonon coupling


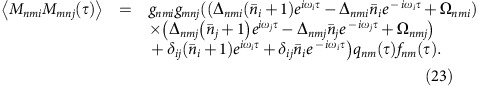


Here


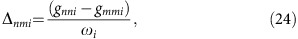



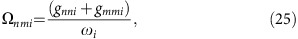



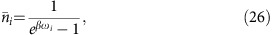



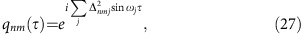



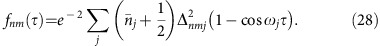


Note that 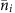 is the Bose population of vibrational normal mode *i*. If specific vibrational normal modes of the system are driven by an IR pulse, the population of these modes are increased and consequently their overall contribution to the golden-rule rate is enhanced.

### Projected mode approach

For a two-state system, the adiabatic Hamiltonian in equation (1) can be written as


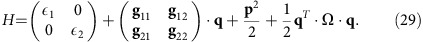


where the **g**_*ij*_ vectors denote the electronic couplings and **q** denotes the normal displacement coordinates. If we imagine that the {**g**_*ij*_} define three unique and non-orthogonal vectors in the *N*-dimensional space of vibrational modes, we can use Schmidt orthogonalization to project a three-dimensional basis embedded in *N*-dimensional that entirely captures the electronic coupling. Our previous work indicates that such decomposition provides a robust description of the short-time dynamics^9^,^10^.

To parametrize equation (29), we start from the diabatic form of Hamiltonian, where only static off-diagonal couplings are kept. Furthermore, if we compute at a minimum of potential energy surface (PES), one diagonal **g** can be eliminated, and we get


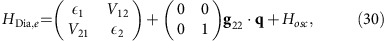


where we assume **g**_22_ is the gradient of the second state's PES at the minimum of the first state's PES, and the off-diagonal coupling *V*_12_ can be approximated by diabatic coupling computed from any diabatization method. The two Hamiltonians can be related by unitary transformation resulting in


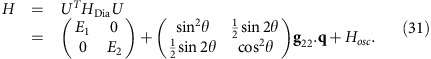


Once we have *H* into this form, we can begin to define a reduced set of modes that capture the electronic coupling.

It is crucial to notice that the vectors given in equation (31) are not linearly independent and we can not generate a reduced subspace as before. Consequently, special care must be taken to generate the reduced subspace. To do so, we use an iterative approach taking the normalized vector **v**_1_=**g**_22_/|*g*_22_| as a starting point. We initialize each step indexed by *k*, by defining a projection operator


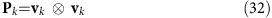


and its complement **Q**_*k*_=**I**−**P**_*k*_. **P**_*k*_ is the projection operator for the *k*th mode. We also construct


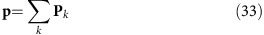


as the total projection operator for all *k*≤*N* modes. We then project the hessian matrix **Ω** into each subspace *viz.*


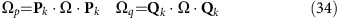


and diagonalize each to obtain eigenvalues and eigenvectors {*α*_*p*_, **M**_*p*_} and {*α*_*q*_, **M**_*q*_} respectively. As above, **Ω**_*p*_ and **Ω**_*q*_ are *N* × *N* matrices. The first set will have a single non-trivial eigenvalue and the second set will have *N*−*k* non-trivial eigenvalues. As above we collect the non-trivial eigenvectors associated with each to form the orthogonal transformation matrix


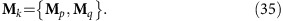


and again transform the full hessian **Ω** into this new vector space to form the *N* × *N* matrix **Ω′**. At each step in the iteration, the transformed hessian, **Ω′** is in the form of a *k* × *k* tridiagonal submatrix in the upper-left part of the matrix and a diagonal submatrix in the lower-right. For example, after *k*=3 iterations, one has a Hessian matrix of the form:


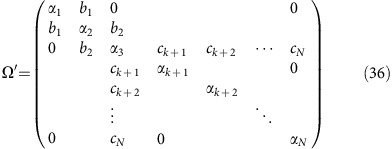


We note that only the *k*th mode is coupled to the *N*−*k* remaining modes. Since all of the transformations are orthogonal, diagonalizing **Ω′** at any point returns the original Hessian matrix.

To continue iterating, we take the *k*th row of **Ω′** and zero the first *k* elements


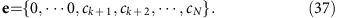


This is the coupling between the upper tridiagonal block and the lower diagonal block. We thus obtain a new vector


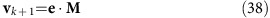


which is then reintroduced into the iteration scheme.

At any point along the way, we can terminate the iteration and obtain a reduced set of couplings. This approach is analogous to the ‘power method' for finding the largest eigenvector of a matrix; consequently, it converges first upon the vector with the largest electron/nuclear coupling–which we refer to as the ‘primary mode'. After *k*-steps, the final electron–phonon couplings are then obtained by projecting the original set of couplings (in the normal mode basis) into the final vector space.

For the first iteration, **v**_1_ is parallel to the bare electron–phonon coupling vector *g*_22_ and the associated frequency is **v**_1_·Ω·**v**_1_. The subsequent iterations introduce corrections to this via phonon–phonon coupling. For example, for the *k*=3 iteration, we would determine the active vector space in terms of the upper-left 3 × 3 block of the matrix in equation (36).


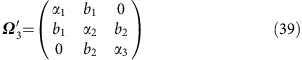


Diagonalizing **Ω**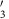 returns a set of frequencies and associated eigenvectors which are then used to compute the electron–phonon couplings in this reduced active space. After *N*−1 iterations, **Ω′** is a fully tridiagonal matrix and diagonalizing this returns the original normal mode basis.

### Data availability

Source code for computing rates and performing the mode analysis are available in the Supporting Information. Quantum chemistry log files will be archived for at least 3 years following the publication date of the paper and can be available upon request to the corresponding author. Request for materials should be directed towards JW at the University of Sheffield.

## Additional information

**How to cite this article:** Yang, X. *et al*. Identifying electron transfer coordinates in donor-bridge-acceptor systems using mode projection analysis. *Nat. Commun.* **8,** 14554 doi: 10.1038/ncomms14554 (2017).

**Publisher's note**: Springer Nature remains neutral with regard to jurisdictional claims in published maps and institutional affiliations.

## Supplementary Material

Supplementary Information Supplementary Note 1

## Figures and Tables

**Figure 1:**
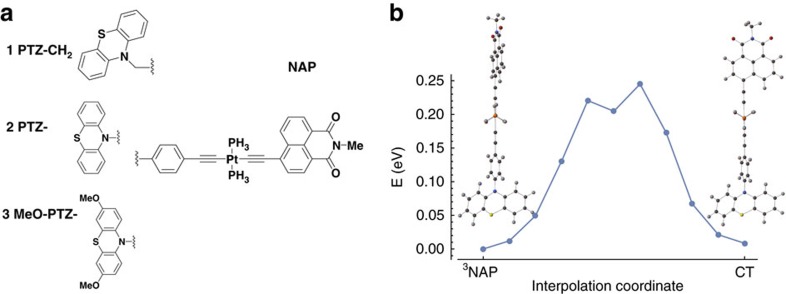
Chemical structures and energies. (**a**) Chemical structures of the donor (D), bridge (-Pt-), acceptor (NAP) complexes considered here. (**b**) Triplet energy along a linear interpolation coordinate connecting the ^3^NAP minimum energy geometry and the CT minimum energy geometry.

**Table 1:** Driving force **Δ** *G*°, reorganization energy *λ*, diabatic coupling *V*, mean diabatic coupling 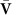 and 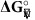 (driving force calculated with 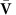 ) for different transitions.

	^**3**^**NAP geom. (0 eV)**		**CT geom. (0.818 eV)**	
	**CSS→**^**3**^**NAP**	**CT→**^**3**^**NAP**	**CT→**^**3**^**NAP**	**CT→CSS**
Δ*G*° (eV)	0.414	−0.913	−0.781	−0.20
*λ* (eV)	1.01	0.271	1.38	1.08
*V*(eV)	2.56E-4	0.345	1.34E-2	9.22E-3
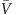 (eV)	6.34E-2	0.106	0.106	0.192
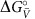 (eV)	0.414	−0.851	−0.770	NA

**Table 2:** Comparison between experimental and computed state-to-state transition rates for PTZ.

	**CT geom.**		^**3**^**NAP geom.**	
**Rates (ps**^**−1**^**)**	**CT→CSS**	**CT→**^**3**^**NAP**	**CT→**^**3**^**NAP**	**CSS→**^**3**^**NAP**
Exp.	0.0879	0.097	0.097	1.84E-3
Marcus	0.846	0.2043	1002.82	8.250E-11
Marcus (mean V)	365.7	12.75	95.23	5.04E-6
TCLME	0.725	0.0562	12.89	3.022E-8
TCLME+PLM	0.627	0.0488	21.6	0.500E-4
TCLME (mean V)	–	2.79	8.931	1.51E-3

The experimental rates for each process are obtained from (ref. 6) the 3-ps lifetime of the CT state, and the relative yields of CT->CSS, CT->3NAP and CT->ground state competing processes.
