# Supplementary material for: Identifying electron transfer coordinates in donor-bridge-acceptor systems using mode projection analysis
Source: Nat Commun. 2017 Feb 24;8:14554. doi: 10.1038/ncomms14554 (PMC5333094; doi:10.1038/ncomms14554)
Supplement: Supplementary Information — Supplementary Note 1 [file ncomms14554-s1.pdf]

SUPPLEMENTARY NOTE 1

Sample codes

The codes below are for the computation of rate constants and projected modes, written in *Mathematica* (v10). The input is taken from a QChem output file.

Helper functions

```
ReadRotM[filename_]: = Module[{stream, RotMat},
stream = OpenRead[filename];
RotMat = Transpose[Partition[ToExpression[Last[Transpose[StringSplit[FindList[filename, "diabatic
    RotMatrix" ]]]]],  2]];
Close[stream];
Return[RotMat]]


ReadEne[filename_]: = Module[{stream, e1, e2},
stream = OpenRead[filename];
e1 = ToExpression[Last[StringSplit[Find[stream, "showmatrix adiabatH(0, 0)"]]]]*27.211396132;
e2 = ToExpression[Last[StringSplit[Find[stream, "showmatrix adiabatH(1, 1)"]]]]*27.211396132(*in eV*);
Close[stream];
Return[{e1, e2}](* in hartree *)]


ReadNormalModes[filename_](* for QChem*):= Module[{stream, NAtom, NMode, NBlock, Freq, ForceConst,
     RedMass, IRActive, IRIntens, RamanActive, NormalModeCoord, IAn, AtomicMass, tmp},
  Freq = {};
  ForceConst = {};
  RedMass = {};
  IRActive = {};
  IRIntens = {};
  RamanActive = {};
  NormalModeCoord = {};
  IAn = {};
```

```
AtomicMass = {};

stream = OpenRead[filename];
NMode = ToExpression[Last[Last[StringSplit[FindList[stream, "Mode"]]]]];
NBlock = If[IntegerQ[#], #, Ceiling[#]]&[NMode/3];
NAtom = (NMode + 6)/3;
Close[stream];

stream = OpenRead[filename];
Find[stream, "Raman Active"];
Read[stream];
tmp = Table[First[StringSplit[Read[stream, String ]]], {NAtom}];
AtomicMass = QuantityMagnitude[ElementData[#, "AtomicWeight"] & /@ tmp];
IAn = ElementData[#, "AtomicNumber"] & /@ tmp;
Close[stream];

stream = OpenRead[filename];
Do[
 Find[stream, "Mode"];
 AppendTo[Freq, ToExpression[Drop[StringSplit[Read[stream, String]], 1]]];
 AppendTo[ForceConst, ToExpression[Drop[StringSplit[Read[stream, String]], 2]]];
 AppendTo[RedMass, ToExpression[Drop[StringSplit[Read[stream, String]], 2]]];
 AppendTo[IRActive, Drop[StringSplit[Read[stream, String]], 2]];
 AppendTo[IRIntens, ToExpression[Drop[StringSplit[Read[stream, String]], 2]]];
 AppendTo[RamanActive, Drop[StringSplit[Read[stream, String]], 2]];
 Read[stream];
 NormalModeCoord = Join[NormalModeCoord, ToExpression[Transpose[Table[Partition[Rest[StringSplit
     [Read[stream, String]]], 3], {NAtom}]]]];
 , {i, 1, NBlock}];
Close[stream];
```

```
  {Freq, ForceConst, RedMass, IRActive, IRIntens, RamanActive} = Flatten /@ {Freq, ForceConst,
      RedMass, IRActive, IRIntens, RamanActive};
  Return[{NAtom, NMode, AtomicMass, Freq, RedMass, NormalModeCoord}]]


ReadGradient[filename_, NAtom_] := Module[{stream, grad, NMode, NBlock, tmp},
  grad = {};
  NBlock = If[IntegerQ[#], #, Ceiling[#]]&[NAtom/6];


  stream = OpenRead[filename];
  Find[stream, "Gradient of the state energy (including CIS Excitation Energy)"];
   Do[
    Read[stream];
    grad = Join[grad, Transpose[Table[ToExpression[Rest[StringSplit[Read[stream, String]]]], {3}]]];
    , {i, 1, NBlock}];
  Return[grad]]
```

```
A_⊗B_:= Outer[Times, A, B]
(* Project1by1 sorts phono modes into a 1 by 1 hierarchical form. Only works for one mode case, for
     general 3 by 3 case it should be modified. *)
Project1by1[gg_, Hess_]: = Module[
{S, P, Q, PHP, QHQ, L, ωc, ωck, ωb, Mc, Mck, Mb, np, ns, Sinv, G, p, k, g},
(* g should be 1-D, namely, ns = 1 *)
{ns, np} = Dimensions[gg];
g = gg;
ωc = {};
Mc = {};
p = ConstantArray[0, {np, np}];


Do[{
S = Table[g[[i]].g[[j]], {i, ns}, {j, ns}];
```

```
Sinv = Inverse[S] ;
(* define projection operators *)
P = Sum[Sinv[[i, j]] g[[ i ]]\[ CircleTimes]g[[ j ]], {i, ns}, {j, ns}] ;
p = p+P; (*P = P1 + P2 + ...*)
Q = IdentityMatrix[np]−p;
PHP = P.Hess.P ;
QHQ = Q.Hess.Q ;
{ωck, Mck} = Chop[Transpose[Take[Sort[Transpose[Eigensystem[PHP]]], −ns]], 10^−8];
{ωb, Mb} = Chop[Transpose[Take[Sort[Transpose[Eigensystem[QHQ]]], ns+k−1−np]], 10^−8];
ωc = Chop[Join[ωc, ωck], 10^−8];
Mc = Chop[Join[Mc, Mck], 10^−8];
ωall= Join[ωc, ωb]//Chop;
M = Join[Mc, Mb]//Chop ;
g = Chop[{Join[ConstantArray[0, k], ( M.Hess.Transpose[M])[[k]][[k+1;;np ]]]. M}, 10^−8]; (* Define nth
    mode = (0, 0, 0, ..., γk+1, γk+2, ..., γk+N) *)
Gnew = M.gg[[1]];
}, {k, 1, np−1}];
](* The kth step gives k*k tridiagonal submatrix on lefttop , diagonal N−k on rightbottom and only the
    kth mode is coupled to the N−k bath modes *)
```

```
NMode[n_, hess_, M_, g_]: = Module[{h, gn, Mnm, ωprime, Gn},
h = Take[(M.hess.Transpose[M]), {1, n}, {1, n}];
{ωprime, Mn} = Transpose[Sort[Transpose[Eigensystem[h]]]];
gn = Join[Take[g, n], ConstantArray[0, n−Length[Take[g, n]]]];
Gn = Mn.gn;
Return[{√ωprime, Gn}]]
```

Initialization

- ## Load Core routines

```
SetDirectory[NotebookDirectory[]];
```

```
Needs["PhysicalConstants`"];  (* this package is outdated ... but highly useful ! *)
```

- # Conversion factors

```
hartree = 27.211396132 ElectronVolt(* eV *);
Bohr = 0.5291772108 Angstrom;
ℏ = hbar = PlanckConstantReduced;
```

- # Read in files

```
name = {"c13ea", "c13ee", "c14ea", "c14ee", "d26ae", "d26ea", "d26ee", "d27ae", "d27ea", "d27ee"
    , "mmm"};
path = "qchemout/"<>#<>".rtf"&/@name;
```

```
kkk = 4;  (* in this case we are looking at c14ee *)
RotMat = ReadRotM[path[[kkk]]];
Ene = ReadEne[path[[kkk]]];
{NAtom, dof, AtomicMass, AngFreq, RedMass, NormMode} = ReadNormalModes[path[[kkk]]];
Grads = ReadGradient[path[[kkk]], NAtom];
ω = FreqeV = Table[Convert[2Pi SpeedOfLight AngFreq[[i]]Centimeter^-1 PlanckConstantReduced,
    ElectronVolt]/ElectronVolt, {i, 1, dof}];
Freq = Table[Convert[2Pi SpeedOfLight AngFreq[[i]]Centimeter^-1 , Second^-1], {i, 1, dof}];
NormModeInMWC = Table[Table[NormMode[[m, i, j]]√(AtomicMass[[i]])/√(RedMass[[m]])//
    Simplify//Chop, {i, NAtom}, {j, 3}], {m, 1, dof}];
Force = Table[Sum[NormModeInMWC[[m]][[i, j]]*Grads[[i, j]]/√(AtomicMass[[i]])//Simplify//Chop,
    {i, 1, NAtom}, {j, 1, 3}], {m, 1, dof}];
GGG = CouplingConstant = Table[Convert[Force[[m]]hartree/Bohr
    √(PlanckConstantReduced/(2Freq[[m]]AMU)), ElectronVolt]/.ElectronVolt->1//Simplify//
    Chop, {m, 1, dof}];
G = G0 = Table[({
 {RotMat[[1, 2]]^2, RotMat[[2, 2]] RotMat[[1, 2]]},
 {RotMat[[2, 2]] RotMat[[1, 2]], RotMat[[2, 2]]^2}
```

```
})*CouplingConstant[[m]]//Simplify//Chop, {m, 1, dof}];
```

Mode Analysis Routines

- ## Analyze n projected modes

```
ModeAnalysis[n_]: = Module[{ωprime},
Clear[imax];
imax = n;
{ωprime, g} = NMode[n, hmat, M, Gnew];
Clear[ω, G];
ω = Take[ωprime, imax];
G = Table[({
 {RotMat[[1, 2]]^2, RotMat[[2, 2]] RotMat[[1, 2]]},
 {RotMat[[2, 2]] RotMat[[1, 2]], RotMat[[2, 2]]^2}
})*g[[m]]//Simplify//Chop, {m, 1, imax}];

v12tab[kkk][imax] = Table[{t, V[1, 2, t]}, {t, 0, nstep*tstep, tstep}];
v21tab[kkk][imax] = Table[{t, V[2, 1, t]}, {t, 0, nstep*tstep, tstep}];
v12[kkk][imax]= Interpolation[v12tab[kkk][imax]];
v21[kkk][imax]= Interpolation[v21tab[kkk][imax]];
b12tab[kkk][imax] = Table[{t, Bd[1, 2, t]}, {t, 0, nstep*tstep, tstep}];
b21tab[kkk][imax] = Table[{t, Bd[2, 1, t]}, {t, 0, nstep*tstep, tstep}];
b12d[kkk][imax]= Interpolation[b12tab[kkk][imax]];
b21d[kkk][imax]= Interpolation[b21tab[kkk][imax]];]
```

- ## Calculation of Correlation Functions

```
nstep = 3000;
tstep = 0.14;
imin = 1;
imax = dof;
```

```
Temperature = 298 Kelvin;

ℏ = hbar= 6.582122*10^(−4)(* eV/ps^−1 =Convert[PlanckConstantReduced /10^−12 /Second,
    ElectronVolt]*);
β = Convert[1/(Temperature BoltzmannConstant), ElectronVolt^−1]/.ElectronVolt−>1;
Clear[ϵ];
ϵ[n_]: = Ene[[n]]−Sum[(G[[i, n, n]]^2/ω[[i]]) , {i , imin, imax}];
nu[i_]: = 1/(Exp[β*ω[[i]]]−1);

Δ[n_, m_, i_]: = (G[[i, n, n]]−G[[i , m, m]])/ω[[i]];
Ω[n_, m_, i_]: = (G[[i, n, n]]+G[[i , m, m]])/ω[[i]];
f[n_, m_, τ_]: = Exp[−2*Sum[(nu[i]+0.5)*(Δ[n, m, i])^2*(1−Cos[ω[[i]]*τ]), {i, imin, imax}]];
q[n_, m_, τ_]: = Exp[I*Sum[(Δ[n, m, i])^2*Sin[ω[[i]]*τ], {i, imin, imax}]];
o[n_, m_, τ_]: = (Sum[G[[i, n, m]]*(Δ[n, m, i]*(nu[i]+1)Exp[I*ω[[i]]*τ]−Δ[n, m, i]*nu[i]*Exp[−I*ω
    [[i]]*τ]+Ω[n, m, i]), {i, imin, imax}])^2+Sum[G[[i, n, m]]*G[[i, m, n]]*((nu[i]+1)Exp[I*ω[[i]]*τ]+
    nu[i]Exp[−I*ω[[i]]*τ]), {i, imin, imax}];
V[m_, n_, τ_]: = 2*f[n, m, τ]*Re[Exp[−I*(ϵ[n]−ϵ[m])*τ]*q[n, m, τ]*o[n, m, τ]];
Bd[m_, n_, τ_]: = f[n, m, τ]*q[n, m, τ]*o[n, m, τ];
```

Get G and [Omega]

```
hess = FreqeV^2;
hmat = DiagonalMatrix[hess];
Project1by1[{CouplingConstant}, hmat]; (*This gives a fully sorted tridiagonal matrix. You may
    encouter singular matrix error for certain molecules, c14ee for example, because some
    couplings are zero. Manually abort it and continue.*)
```

```
ModeAnalysis[dof](*Exact calculation *)
```

```
ModeAnalysis[1](*Do however many modes you like.*)
ModeAnalysis[4]
ModeAnalysis[7]
```

```
Do[ModeAnalysis[i], {i , 5, 20, 5}]
```

Rate

```
φ12 = Table[NIntegrate[v12[kkk][dof][τ], {τ, i*tstep, (i+1)*tstep}], {i , 0, nstep−1}];
φ21 = Table[NIntegrate[v21[kkk][dof][τ], {τ, i*tstep, (i+1)*tstep}], {i , 0, nstep−1}];
Ww12 = {{0, 0}};
Ww21 = {{0, 0}};
ωω12 = 0;
ωω21 = 0;
Do[ωω12p = ωω12+φ12[[i]];
Ww12 = Append[Ww12, {i*tstep*ℏ, ωω12p}];
ωω12 = ωω12p, {i, nstep}];
Do[ωω21p = ωω21+φ21[[i]];
Ww21 = Append[Ww21, {i*tstep*ℏ, ωω21p}];
ωω21 = ωω21p, {i, nstep}];
W12t= Interpolation[Ww12];
W21t= Interpolation[Ww21];
vm12 = Mean@Take[Table[Ww12[[i]][[2]], {i, 1, nstep }], −30];
vm21 = Mean@Take[Table[Ww21[[i]][[2]], {i, 1, nstep }], −100];
W12 = Function[Piecewise[{{W12t[#], 0< = #< = nstep*tstep}, {vm12 , #>nstep*tstep*ℏ}}]];
W21 = Function[Piecewise[{{W21t[#], 0< = #< = nstep*tstep}, {vm21 , #>nstep*tstep*ℏ}}]];
Print["Golden rule rate =", vm21/ℏ*10^12, "s^-1"];
```

Correlation function

```
Plot[{
  Re[b21d[kkk][dof ][.001 t/ℏ]],
  Re[b21d[kkk][1][.001 t/ℏ]], Re[b21d[kkk][4][.001 t/ℏ]],
  Re[b21d[kkk][7][.001 t/ℏ]]},
{t, 0, 10},
PlotRange −> All,
```

```
AspectRatio -> 1,
PlotStyle -> {{Black, Thick, Dashing[0.01]}, Blue, Green, Red, Purple},
LabelStyle -> 16,
AxesLabel -> {"t/fs", "Re(C12(t)))"},
PlotLegends -> Placed[{"Exact", "1 Mode", "4 Modes", "7 Modes", "7 Modes"}, Right],
Epilog -> {Style[Text[name[[kkk]], {9, -2.5*10^-7}], 24]}]
```
